# Noise-Adaption Extended Kalman Filter Based on Deep Deterministic Policy Gradient for Maneuvering Targets

**DOI:** 10.3390/s22145389

**Published:** 2022-07-19

**Authors:** Jiali Li, Shengjing Tang, Jie Guo

**Affiliations:** School of Aerospace Engineering, Beijing Institute of Technology, Beijing 100081, China; 3120185066@bit.edu.cn (J.L.); tangsj@bit.edu.cn (S.T.)

**Keywords:** maneuvering target, noise adaption, maneuver detection, Dempster-Shafer evidence theory, deep deterministic policy gradient

## Abstract

Although there have been numerous studies on maneuvering target tracking, few studies have focused on the distinction between unknown maneuvers and inaccurate measurements, leading to low accuracy, poor robustness, or even divergence. To this end, a noise-adaption extended Kalman filter is proposed to track maneuvering targets with multiple synchronous sensors. This filter avoids the simultaneous adjustment of the process model and measurement model without distinction. Instead, the maneuver detection based on the Dempster-Shafer evidence theory is constructed to achieve the reliable distinction between unknown maneuvers and inaccurate measurements by fusing multi-sensor information, which effectively improves the robustness of the filter. Moreover, the adaptive estimation of the process noise covariance is modeled by a Markovian decision process with a proper reward function. Deep deterministic policy gradient is designed to obtain the optimal process noise covariance by taking the innovation as the state and the compensation factor as the action. Furthermore, the recursive estimation of the measurement noise covariance is applied to modify a priori measurement noise covariance of the corresponding sensor. Finally, the fusion algorithm is developed for the global estimation. Simulation experiments are carried out in two scenarios, and simulation results illustrate the feasibility and superiority of the proposed algorithm.

## 1. Introduction

With the development of high maneuvering targets, such as hypersonic aircrafts and missiles, maneuvering target tracking has recently drawn a lot of attention [1,2]. During the past few decades, lots of tracking methods have been developed and investigated for maneuvering targets [3,4,5]. Among the existing target tracking approaches, designing an appropriate model for the target maneuvering motion is regarded as an important part of decreasing the tracking errors caused by the model mismatch. Therefore, many maneuver models were proposed, such as constant acceleration (CA) [6], Singer model [7], current statistical model (CS) [8]. In contrast to single model methods, the interactive multiple model (IMM) algorithm [9,10] has been widely applied because of its adaptive capability for maneuvering target tracking problems. By mapping the motion mode to a model set, IMM performs the parallel filter for each model. Furthermore, based on the residual information and a priori information, the state estimation results of each model are weighted and synthesized to accurately achieve the maneuvering target tracking. Considering the limited performance of IMM based on the single platform, Wang et al. [11] proposed a multi-platform maneuvering target tracking algorithm based on IMM and the best linear unbiased estimate filter. Moreover, an adaptive interacting multiple model algorithm based on information-weighted consensus was proposed [12], which presented better adaptability and accuracy than the classical IMM. To cope with the miss detections and false alarms in a low-observable environment, a state-dependent IMM based on the particle filter was presented [13]. However, the above IMM algorithms usually use a fixed set of models, so a large number of model sets is required when solving the problem of maneuvering target tracking. To alleviate this problem, the variable structure multiple model (VSMM) method has been proposed [14]. Furthermore, a VSMM version of the consensus filter was presented [15], where the expected mode augmentation was introduced to guarantee the model set adaption. In addition, the particle swarm optimization technique was utilized to improve VSMM [16]. However, for maneuvering target tracking in practice, multiple models cannot fully cover the modes of the target’s motion, which may degrade the tracking performance. Additionally, excessive numbers of models also generate large calculations in many practical applications.

On the other hand, the filtering algorithm is regarded as an important issue to cope with maneuvering target tracking problems. Practically, extended Kalman filter (EKF) [17] has been widely applied for many nonlinear systems during the past few years. EKF is developed based on the Kalman filter and introduces the first-order Taylor expansion to approximate the nonlinear function. However, the first-order Taylor expansion of EKF cannot, at times, satisfy the tracking accuracy. To this end, other nonlinear filter algorithms including unscented Kalman filter (UKF) [18,19] and cubature Kalman filter (CKF) [20] were proposed. By adopting the unscented transformation and spherical-radial principle, UKF and CKF can achieve second-order accuracy without calculating the Jacobian matrix. In order to further improve the robustness of the conventional filters, the strong tracking filter (STF) was proposed by Zhou et al. [21,22]. This algorithm introduces the orthogonal principle of residual sequences into the classical EKF to modify the prediction of the covariance matrix with a time-varying fading factor. When the state estimation of EKF deviates from the actual state, the effect of the old observations on the current filter estimation is reduced using the time-variant fading factor whereas the effect of the new observation is increased [23]. Accordingly, the robustness and accuracy of the classical EKF were improved. In order to improve the performance of STF, UKF and CKF were introduced to replace EKF [24,25]. An adaptive strong tracking particle filter algorithm was established by combing STF and the particle filter (PF), which demonstrated that the proposed method could provide better tracking precision than the classical methods [26]. Since the traditional strong tracking filter only considers the first-order Taylor expansion, a fast-strong tracking CKF was proposed [27], which expanded the number of fading factors from one to two. Sequentially, it extracted the third-order term information and achieved second-order accuracy. To implement higher tracking accuracy for the maneuvering target tracking, many improved strong tracking cubature Kalman filter (STCKF), such as strong tracking spherical simplex-radial CKF [28], fifth-degree STCKF [29], Bayesian-based strong tracking interpolatory CKF [30], model-based strong tracking square-root CKF [31], have recently been proposed. In addition, Ma et al. [32] added STF into the sub-filter of IMM to overcome the low tracking accuracy when dealing with maneuvering situations. However, in practical applications, it is found that the detection and tracking ability of the STF method will be reduced when the maneuver of the target is small. As is known, the residuals of range measurements are usually much bigger than the residuals of azimuth angle and elevation angle measurements, which leads STF to different sensitivities of different residual components. To this end, Jiang et al. [33] proposed a residual-normalized strong tracking filter (RNSTF) by designing the weight of the residual components. However, the process noise is not considered.

The aforementioned algorithms adopt the innovation, also known as the predictive residual, to realize maneuver detection and compensation, because the innovation will increase significantly when the model mismatch is caused by the unknown maneuver. However, the sensor signal may be disturbed or blocked due to the influence of a harsh environment, making the statistical characteristics of measurement noise uncertain and time varying. Inaccurate a priori measurement noise covariance can also lead to increased innovation. In that case, the aforementioned algorithms are too sensitive to inaccurate measurements, leading to filter divergence. To solve the problem of inaccurate a priori knowledge, several adaptive Kalman filters (AKF) with noise sensing capability were established [34,35]. Unfortunately, the existing methods only can achieve the second-order estimation accuracy [36,37], and the adaptive KF cannot distinguish between model mismatch and inaccurate a priori measurement noise. In addition, adaptive KFs use the innovation to adjust the process noise and measurement noise simultaneously, which leads to poor stability.

Inspired by the strong representation capability of neural networks [38,39], several filters based on reinforcement learning have been studied. Hu et al. [40] designed an attitude estimator by combining Lyapunov’s method and the deep reinforcement learning algorithm. Tang et al. [41] combined the classic EKF with the deep reinforcement learning algorithm to realize the attitude estimation of the navigation system, which introduced a gain matrix of the residual and took it as the action to learn. Gao et al. [42] proposed an adaptive Kalman filter based on the deep deterministic policy gradient (DDPG) algorithm for ground vehicles, which took the integrated navigation system as the environment to obtain the process noise covariance matrix estimation. However, this adaptive filter takes the change in noise vector as the action to learn, resulting in poor filter stability. Additionally, the process noise covariance matrix of Inertial Navigation System (INS) error model will not experience a great change because it represents the inherent performance of the inertial sensor [42], which is different from the target tracking problem.

Motivated by the above investigation, a noise-adaption EKF is proposed in this paper to address the problem of maneuvering target tracking. Based on multiple synchronous sensors, the maneuver detection is constructed by utilizing Dempster-Shafer (D-S) evidence theory [43] to achieve the fused detection of multiple sensors. A Markovian decision process with a proper reward function is modeled for the adaptive estimation of the process noise covariance, and DDPG is designed to learn the compensation factor and feed it into EKF so that the improved filter can adaptively cope with the unknown maneuver. If the detection declares the occurrence of inaccurate measurement noise, the recursive measurement noise estimation is applied to the corresponding sensor. Finally, the local estimations are fused to obtain the target’s global estimation. When the unknown maneuver and mismatched measurement noise emerge, the proposed filter can correct noise covariance adaptively. Distinct from the aforementioned algorithms, the proposed filter avoids the simultaneous adjustment of process model and measurement model without distinction, which effectively improves the robustness of the filter. Moreover, the application the deep deterministic policy gradient method in solving maneuver target tracking problems is explored in this paper. 

The remainder of this paper is organized as follows: Section 2 introduces mathematical models and the formula of EKF. Section 3 provides the maneuver detection based on D-S evidence theory in detail, and the framework of the noise-adaption EKF is presented. In Section 4, the process noise adaption based on DDPG is designed. Moreover, the recursive measurement noise estimation and the fusion algorithm are completed. Finally, simulation results and conclusions are shown in Section 5 and Section 6, respectively.

## 2. Problem Formulation

The process model of the target is given by

(1) $$x_{k+1}=f\left(x_{k}\right)+w_{k}$$

where k denotes the index of discrete-time. $x_{k}$ represents the state vector, and f(⋅) is the state transition function. The process noise $w_{k}$ is assumed to be zero-mean Gaussian white noise with the covariance matrix $Q_{k}$.

The measurement model of sensors can be represented as

(2) $$z_{k+1}=h\left(x_{k+1}\right)+v_{k+1}$$

where $z_{k+1}$ is the measurement vector, and h(⋅) is the measurement mapping function. $v_{k+1}$ is the measurement noise, whose covariance matrix is $R_{k+1}$.

Since EKF has the advantages of simple algorithm and fast convergence, it has been widely applied in nonlinear systems [17]. Specifically, EKF can be divided into two stages. The first stage is the one-step prediction based on the process model

(3) $${\hat{x}}_{k+1|k}=f\left({\hat{x}}_{k}\right)$$

(4) $$P_{k+1|k}=F_{k+1|k}P_{k|k}F_{k+1|k}^{T}+Q_{k}$$

where ${\hat{x}}_{k|k}$ is the state estimation and $P_{k|k}$ is the covariance matrix, ${\hat{x}}_{k+1|k}$ and $P_{k+1|k}$ are the corresponding predictions. The state transition matrix $F_{k+1|k}$ is defined as the first-order Taylor expansion of the state transition function.

(5) $$F_{k+1|k}={\left.\frac{\partial f\left(x\right)}{\partial x}\right|}_{x={\hat{x}}_{k}}$$

The second stage is the one-step update based on the measurement. The state vector and its covariance matrix are updated as

(6) $$K_{k+1}=P_{k+1|k}H_{k+1}^{T}{\left(H_{k+1}P_{k+1|k}H_{k+1}^{T}+R_{k+1}\right)}^{-1}$$

(7) $${\hat{x}}_{k+1}={\hat{x}}_{k+1|k}+K_{k+1}\left[z_{k+1}-h\left({\hat{x}}_{k+1|k}\right)\right]$$

(8) $$P_{k+1|k+1}=\left(I-K_{k+1}H_{k+1}\right)P_{k+1|k}$$

where $K_{k+1}$ is the gain matrix, and $H_{k+1}$ is defined as

(9) $$H_{k+1}={\left.\frac{\partial h\left(x\right)}{\partial x}\right|}_{x={\hat{x}}_{k+1|k}}$$

As the main prior information of the system, the process noise Q and the measurement noise R are of great significance to the estimation performance and stability of the filter. Q and R represent the confidence degree in models and measurements, respectively. If Q and R are smaller than the true noise distribution, the uncertainty range of the true state is too narrow, resulting in estimation bias. Conversely, if Q and R are larger than the true noise distribution, it may lead to filter divergence. Additionally, inaccurate Q and R can impair the estimation accuracy.

## 3. Maneuver Detection and the Framework

### 3.1. Maneuver Detection Based on D-S Evidence Theory

The key to maneuver detection is to design an appropriate detection strategy to detect the target’s maneuver precisely and timely. As stated in the introduction, the detection depending on the innovation from single-source information is not reliable. To improve the accuracy of the maneuver detection, the maneuver detection with a sliding-window structure based on D-S evidence theory is proposed in this section by introducing multiple synchronous sensors’ information.

D-S evidence theory is a fusion rule established on a nonempty finite space, which includes a limited number of subsets [43]. Through independent observations by sensors, it can fuse observation results and give a joint judgment to improve the confidence and accuracy of events. D-S evidence theory can combine the evidence more intuitively and easily, and it has the expression ability of unknown and uncertain situations by calculating the probability of the set of multiple events.

Consider all the subsets of the finite space Θ as $2^{\Theta }$, including Θ and an empty set Φ, and define the map m:$2^{\Theta }\to [0,1]$, for A⊂Θ, it satisfies

(10) $$\sum_{A\subset \Theta }m\left(A\right)=1$$

(11) $$m\left(\Phi \right)=0$$

where the map $m\left(\cdot \right)$ is called the basic probability assignment function. $m\left(A\right)$ is called the mass function of A, which indicates the degree of confidence for A according to the current observation. 

For the problem of maneuvering target tracking, the target has two movement modes: maneuver or not, so there are two events in the maneuver detection problem: “Maneuver” and “Normal”, denoted as $A_{1}$, $A_{2}$, respectively. However, when the degree of confidence tends to zero, unreasonable fusion results will be obtained, which is always called as Zadeh paradox [43]. To avoid the Zadeh paradox, one more event “uncertainty” is added, denoted as $A_{3}=\left\{A_{1},A_{2}\right\}$, and its confidence degree is set to 0.01.

Suppose there are N sensors applied in the target’s measurement mission, denoted as $S_{i}\left(i=1,2,\ldots ,N\right)$, and the measurement of $S_{i}$ at time k is $z_{i,k}$. The innovation is defined as the difference between actual measurement value and predictive measurement value.

(12) $$\gamma_{i,k+1}=z_{i,k+1}-{\hat{z}}_{i,k+1|k}$$

where ${\hat{z}}_{i,k+1|k}=h_{i}\left({\hat{x}}_{i,k+1|k}\right)$ is the predictive measurement. The innovation satisfies

(13) $$E\left[\gamma_{i,k+1}\right]=0$$

(14) $$E\left[\gamma_{i,k+1}\gamma_{i,k+1}^{T}\right]=H_{i,k+1}P_{i,k+1|k}H_{i,k+1}^{T}+R_{i,k+1}$$

Following this, the detection variable is constructed based on the innovation.

(15) $$\eta_{i,k+1}=\gamma_{i,k+1}^{T}{\left(H_{i,k+1}P_{i,k+1|k}H_{i,k+1}^{T}+R_{i,k+1}\right)}^{-1}\gamma_{i,k+1}$$

Since the innovation is subject to Gaussian distribution, the detection variable is supposed to be subject to $\chi^{2}$ distribution with n degree of freedom, where n is the dimension of the innovation vector. Therefore, the maneuver probability is defined as

(16) $$P_{i,k+1}(\mathrm{Maneuver})=\int_{0}^{\eta_{i,k+1}}\chi_{n}^{2}\left(x\right)dx$$

It can be seen from Equation (16) that, the innovation is considered to be entirely caused by the unknown maneuver, hypothetically. The increase in the innovation indicates bigger maneuver probability. 

Based on the definition of the maneuver probability, the mass function of $S_{i}$ is given as

(17) $$m_{i,k+1}\left(A_{1}\right)=P_{i,k+1}\left(\mathrm{Maneuver}\right)$$

(18) $$m_{i,k+1}\left(A_{2}\right)=1-P_{i,k+1}\left(\mathrm{Maneuver}\right)-0.01$$

(19) $$m_{i,k+1}\left(A_{3}\right)=0.01$$

As mentioned above, the mismatch between the real measurement noise and a priori measurement noise can also lead to the innovation’s increase. As a result, the maneuver probability determined by the innovation is unreliable. A joint detection mechanism is developed by fusing maneuver detection results of multiple synchronous sensors to improve the accuracy and reliability of the maneuver detection. For N independent pieces of evidence with $N_{e}$ events, the fused mass function of $A_{1}$ can be merged by D-S evidence theory as follows

(20) $$\begin{array}{ccc} m_{k+1}\left(A_{1}\right) & = & \frac{\sum_{A_{1}\cap \cdots \cap A_{N_{e}}=A_{1}}m_{1,k+1}\left(A_{1}\right)\cdot m_{2,k+1}\left(A_{2}\right)\cdots m_{N,k+1}\left(A_{N_{e}}\right)}{1-\sum_{A_{1}\cap \cdots \cap A_{N_{e}}=\;\;\;\Phi \;\;\;}m_{1,k+1}\left(A_{1}\right)\cdot m_{2,k+1}\left(A_{2}\right)\cdots m_{N,k+1}\left(A_{N_{e}}\right)}\\ & = & \frac{\sum_{A_{1}\cap \cdots \cap A_{N_{e}}=A_{1}}m_{1,k+1}\left(A_{1}\right)\cdot m_{2,k+1}\left(A_{2}\right)\cdots m_{N,k+1}\left(A_{N_{e}}\right)}{\sum_{A_{1}\cap \cdots \cap A_{N_{e}}\neq \;\;\;\Phi \;\;\;}m_{1,k+1}\left(A_{1}\right)\cdot m_{2,k+1}\left(A_{2}\right)\cdots m_{N,k+1}\left(A_{N_{e}}\right)} \end{array}$$

where $N_{e}=3$ in this paper. 

Similarly, the fused mass function of $A_{2}$ and $A_{3}$ can be acquired, denoted as $m_{k+1}\left(A_{2}\right)$ and $m_{k+1}\left(A_{3}\right)$, respectively. If $m_{k+1}\left(A_{1}\right)\geq m_{k+1}\left(A_{2}\right)$, it means that the target is detected to be maneuvering according to the fusion of multiple sensors at time k. Conversely, the target is not maneuvering when $m_{k+1}\left(A_{1}\right)<m_{k+1}\left(A_{2}\right)$, and the mismatch of the a priori measurement noise is the main reason for the increased innovation.

However, the maneuver detection based on the current time is unstable. Furthermore, the sliding window structure is introduced into the maneuver detection to improve the stability of detection, where the detection depends not only on the current state but also on the neighboring states. 

The size of the sliding window is set as $N_{sw}$, and the final mass function $m_{k+1}^{f}\left(\cdot \right)$ can be calculated by


(21) $$m_{k+1}^{f}\left(A_{1}\right)=\left\{\begin{array}{c} \begin{array}{cc} \frac{\sum_{A_{1}\cap \cdots \cap A_{N_{e}}=A_{1}}m_{k+2-N_{sw}}\left(A_{1}\right)\cdots m_{k}\left(A_{N_{e}}\right)\cdot m_{k+1}\left(A_{N_{e}}\right)}{\sum_{A_{1}\cap \cdots \cap A_{N_{e}}\neq \;\;\;\Phi \;\;\;}m_{k+2-N_{sw}}\left(A_{1}\right)\cdots m_{k}\left(A_{N_{e}}\right)\cdot m_{k+1}\left(A_{N_{e}}\right)} & k+1\geq N_{sw} \end{array}\\ \begin{array}{cc} \frac{\sum_{A_{1}\cap \cdots \cap A_{N_{e}}=A_{1}}m_{1}\left(A_{1}\right)\cdots m_{k}\left(A_{N_{e}}\right)\cdot m_{k+1}\left(A_{N_{e}}\right)}{\sum_{A_{1}\cap \cdots \cap A_{N_{e}}\neq \;\;\;\Phi \;\;\;}m_{1}\left(A_{1}\right)\cdots m_{k}\left(A_{N_{e}}\right)\cdot m_{k+1}\left(A_{N_{e}}\right)} & k+1<N_{sw} \end{array} \end{array}\right.$$

Similarly, the final mass function $m_{k+1}^{f}(A_{2})$ and $m_{k+1}^{f}(A_{3})$ can be obtained. According to the fused detection result, there are three conditions of the tracking problem. (a) When $m_{k+1}^{f}(A_{1})\geq m_{k+1}^{f}(A_{2})$, the target is detected to be maneuvering, and the adaptive process noise estimation needs to be introduced to deal with unknown maneuvers. (b) If $m_{k+1}^{f}(A_{1})<m_{k+1}^{f}(A_{2})$ and $m_{i,k+1}(A_{1})\geq m_{i,k+1}(A_{2})$, the target is not maneuvering, and the mismatch of measurement noise emerges, so the measurement noise adaption of $S_{i}$ is necessary. (c) There are no above two conditions when $m_{k+1}^{f}(A_{1})<m_{k+1}^{f}(A_{2})$ and $m_{i,k+1}(A_{1})<m_{i,k+1}(A_{2})$, so EKF can cope with the target tracking task.

### 3.2. The Framework of the Noise-Adaption EKF

**Figure 1:**
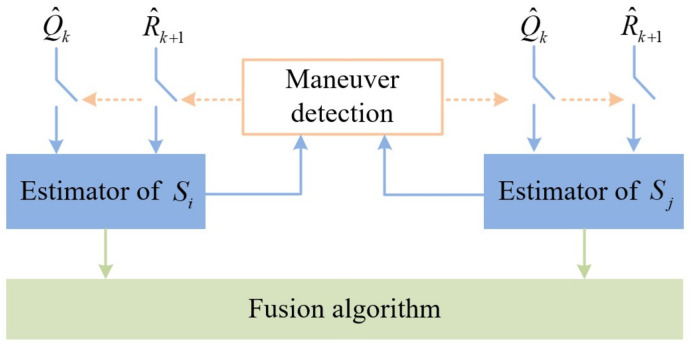
The framework of the noise-adaption EKF.

Based on the above maneuver detection, a noise-adaption EKF is proposed to adaptively cope with the unknown maneuver and inaccurate measurement noise. The framework of the proposed filter is shown in Figure 1.

To achieve the accurate compensation for unknown maneuvers and the inaccurate measurement noise covariance, multi-sensor information undergoes the maneuver detection. Whether or not the process noise adaption or the measurement noise adaption proceeds is determined by the maneuver detection. If the target is detected to be maneuvering, the adaptive process noise is calculated by DDPG and introduced into the filter to cope with the model mismatch caused by the unknown maneuver. If the inaccurate measurement noise covariance is detected to be the main reason for the innovation’s increase, the recursive measurement noise estimation is applied to the corresponding sensor. Sequentially, the local estimations are fused to obtain the global estimation of the target. When the unknown maneuver or mismatched measurement noise emerge, the proposed filter can estimate the maneuvering target’s state with high performance.

## 4. Noise-Adaption EKF for Maneuvering Targets

### 4.1. Process Noise Adaption Based on DDPG

For the problem of maneuvering target tracking, the main task is to find a tracking strategy to estimate the target’s station rapidly and accurately. However, the accurate mathematical models of non-cooperative targets cannot be obtained. When the target is maneuvering, the estimation error increases due to the model mismatch. To cope with the unknown maneuver, an effective method is to adaptively estimate the process noise covariance online. To this end, a process noise adaption method based on DDPG is proposed in this section. Through its self-learning and optimization capabilities, DDPG algorithm can adaptively cope with the unknown maneuver.

**Figure 2:**
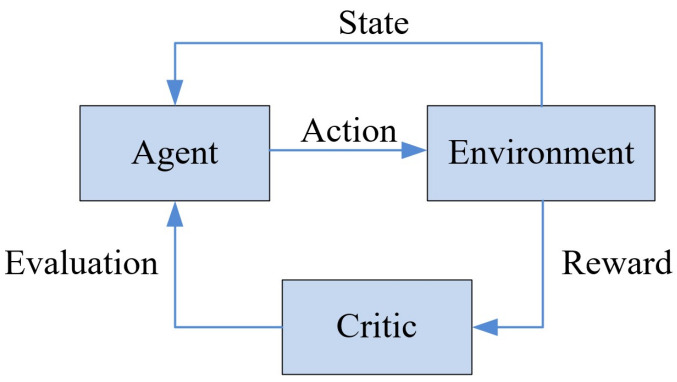
The basic framework of DDPG.

DDPG is known as one of the reinforcement learning algorithms for the continuous action strategy. The interactive learning process of DDPG is formalized as the Markov decision process (MDP), which can be defined by four elements: the state $S=\left\{s_{1},s_{2},\cdots \right\}$, the action $A=\left\{a_{1},a_{2},\ldots \right\}$, the reward $R=\left\{r_{1},r_{2},\cdots \right\}$, and the state transition probability $P\left(s_{k+1}|s_{k},a_{k}\right)$. The framework of DDPG is shown in Figure 2. The agent generates an action to interact with the environment at first, and then under the joint effects of action and environment, a new state is generated, and the environment gives a reward to the critic. Afterwards, the critic calculates the action-value function to evaluate the given policy, then the action policy is further optimized according to the evaluation results.

The adaptive estimation of the process noise covariance is defined as

(22) $${\hat{Q}}_{k}=\lambda_{k}{\hat{Q}}_{k-1}$$

where ${\hat{Q}}_{k}$ is the adaptive process noise covariance, and its initial value is the a priori process noise covariance matrix. $\lambda_{k}>1$ is the compensation factor. This adaptive estimation form is more robust due to fewer parameters.

Correspondingly, the prediction of the covariance matrix in Equation (4) is updated as

(23) $$P_{k+1|k}=F_{k+1|k}P_{k|k}F_{k+1|k}^{T}+{\hat{Q}}_{k}$$

Following by this, the problem of designing the maneuvering tracking strategy is transformed into the determination of the compensation factor, which can be described as an MDP. DPPG algorithm is designed to learn the compensation factor, so the action is defined as

(24) $$a_{k}=\lambda_{k}$$

The innovation reflects the degree of model mismatch. The larger the innovation, the larger the model error, and the compensation factor needs to be increased accordingly. Therefore, the agent state is defined as the filter’s innovation in Equation (12), which contains three-dimensional innovation: range, azimuth angle and elevation angle, i.e.,

(25) $$s_{k}=\gamma_{k}^{T}={\left[\gamma_{k,r},\gamma_{k,\beta },\gamma_{k,\varepsilon }\right]}^{T}$$

When the environment, i.e., the filter, receives an action, a sufficient reward mechanism is required to evaluate the filter performance under the current action. Therefore, the filter residual is taken as the evaluation index, which is expressed as

(26) $$\varphi_{k}=z_{k}-{\hat{z}}_{k}=z_{k}-h\left({\hat{x}}_{k}\right)$$

In practice, the residuals of range measurements are usually much bigger than the residuals of azimuth angle and elevation angle measurements in practice, which leads to different sensitivity to different residual components. To make the filter equally sensitive to residual elements, a weighting matrix W is introduced for normalizing the residuals.

(27) $$W=\left[\begin{array}{ccc} 1/\sigma_{r} & 0 & 0\\ 0 & 1/\sigma_{\beta } & 0\\ 0 & 0 & 1/\sigma_{\varepsilon } \end{array}\right]$$

where $\sigma_{r}$, $\sigma_{\beta }$, and $\sigma_{\varepsilon }$ represents the measurement errors’ standard deviation of range, azimuth angle, and elevation angle, respectively.

Therefore, the reward function is established as follows

(28) $$r_{k}=tr[(W\varphi_{k}){\left(W\varphi_{k}\right)}^{T}]$$

where tr[⋅] is the trace operation. 

In DDPG algorithm, two neural networks including the actor network and the critic network, are utilized to approximate the action function and the action-value function. Denote $A^{\mu }(s_{k})$ as the action function, which is parameterized by μ. It maps from state to action, i.e., $a_{k}=A^{\mu }(s_{k})$. Moreover, the action-value function is denoted as $Q^{\omega }(s_{k},a_{k})$, which is parameterized by ω. Additionally, to enhance the convergence of training, one target actor network and one target critic network are introduced into DDPG, denoted as $A^{\mu ’}(s_{k})$ and $Q^{\omega ’}(s_{k},a_{k})$, respectively.

The optimal action policy is realized by maximizing the expected total reward

(29) $$\mu =\underset{\mu }{\arg\max}J(A^{\mu })$$

The expected total reward is defined as

(30) $$J(A^{\mu })=E\left[Q^{\omega }(s,a){|}_{s=s_{k},a=A^{\mu }(s_{k})}\right]$$

Therefore, the actor function is optimized toward the gradient of the expected total reward

(31) $$\nabla_{\mu }J(A^{\mu })=\nabla_{\mu }A^{\mu }(s_{k})\nabla_{a_{k}}Q^{\omega }(s_{k},a_{k})$$

Similarly, the action-value function is optimized by minimizing the loss function

(32) $$\omega =\underset{\omega }{\arg\min}L(\omega )$$

The temporal difference error $\delta_{k}$ is utilized to establish the loss function

(33) $$L(\omega )=\delta_{k}^{2}={\left[r_{k}+\xi Q^{\omega ’}(s_{k+1},A^{\mu ’}(s_{k+1}))-Q^{\omega }(s_{k},A^{\mu }(s_{k}))\right]}^{2}$$

where $r_{k}$ is the reward, and ξ is the discounting factor.

The actor-value function is optimized toward the gradient of the loss function

(34) $$\nabla_{\omega }L(\omega )=-2\delta_{k}\nabla_{\omega }Q^{\omega }(s_{k},a_{k})$$

To improve the stability in the DDPG training process, there is a replay memory buffer ℛ to store the training samples. At every training step, training samples with a size $N_{sample}$ are utilized to train the actor and critic networks. The current transition experience $\left(s_{k},a_{k},r_{k},s_{k+1}\right)$ will be added into the buffer to replace the oldest one.

The actor network is updated by the Adam optimizer

(35) $$\mu_{k+1}=f_{Adam}(\mu_{k},\nabla_{\mu }J(A^{\mu }))$$

(36) $$\nabla_{\mu }J(A^{\mu })=\frac{1}{N_{sample}}\sum_{i=1}^{N_{sample}}\nabla_{\mu }A^{\mu }(s_{i})\nabla_{a_{k}}Q^{\omega }(s_{i},a_{i})$$

Similarly, the critic network is updated using the negative gradient of the loss function

(37) $$\omega_{k+1}=f_{Adam}(\omega_{k},\nabla_{\omega }L(\omega ))$$

(38) $$\nabla_{\omega }L(\omega )=\frac{1}{N_{sample}}\sum_{t=1}^{N_{sample}}\delta_{i}\nabla_{\omega }Q^{\omega }(s_{i},a_{i})$$

At the end of each sample training, the two target networks are soft updated as

(39) $$\mu {’}_{k+1}=\tau \mu_{k+1}+(1-\tau )\mu {’}_{k}$$

(40) $$\omega {’}_{k+1}=\tau \omega_{k+1}+(1-\tau )\omega {’}_{k}$$

where τ is the updating rate.

**Figure 3:**
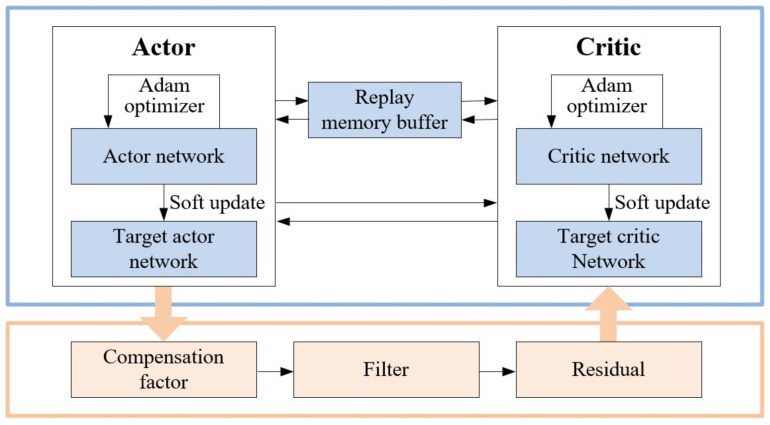
The implementation framework of the process noise adaption based on DDPG.

The implementation framework of the adaptive estimation of process noise based on DDPG is shown in Figure 3.

The process noise adaption based on DDPG is summarized in Algorithm 1.
**Algorithm 1** The process noise adaption based on DDPG1 Initialize the parameters of the actor network and critic network2 Initialize target networks by copying the actor and critic network3 Initialize the replay memory buffer ℛ
**For each episode, perform the following steps**4 Initialize the estimation state and its covariance matrix
  **For each timestep, perform the following steps**
5   Generate an action based on the actor network and the current state $a_{k}=A^{\mu }(s_{k})+{𝒩}_{k}$, where the random noise is generated by Ornstein-Uhlenbeck process6   Execute the action, i.e., the compensation factor in the filter to obtain a new state $s_{k+1}$ and a new reward $r_{k}$
7   Store the sample $\left(s_{k},a_{k},s_{k+1},r_{k}\right)$ in the buffer ℛ
8   Randomly select $N_{Sample}$ samples from the buffer9   Calculate the temporal difference error $\delta_{p}$ of each sample
   $\delta_{p}=r_{p}+\xi Q^{\omega^{’}}(s_{p+1},A^{\mu^{’}}(s_{p+1}))-Q^{\omega }(s_{p},A^{\mu }(s_{p}))$
10   Calculate the policy gradient
   $\nabla_{\mu }J(A^{\mu })=\frac{1}{N_{Sample}}\sum_{p=1}^{N_{Sample}}\nabla_{\mu }A^{\mu }(s_{p})\nabla_{a_{t}}Q^{\omega }(s_{p},a_{p})$
11   Update the actor network by Adam optimizer:
   $\mu_{k+1}=f_{Adam}(\nabla_{\mu }J(A^{\mu }))$
12   Update the critic network
   $\nabla_{\omega }ℒ(\omega )=\frac{1}{N_{Sample}}\sum_{p=1}^{N_{Sample}}-2\delta_{p}\nabla_{\omega }Q^{\omega }(s_{p},a_{p})$

   $\omega_{k+1}=f_{Adam}(\omega_{t},\nabla_{\omega }ℒ(\omega ))$
13   Update the two target networks by soft update
   $\mu {’}_{k+1}=\tau \mu_{k+1}+(1-\tau )\mu {’}_{k}$

   $\omega {’}_{k+1}=\tau \omega_{k+1}+(1-\tau )\omega {’}_{k}$

 **End timestep**
**End episode**

### 4.2. Recursive Measurement Noise Estimation

When the sensor’s maneuver detection result is inconsistent with the fused maneuver detection result, the corresponding sensor’s measurement model is considered not to match a priori knowledge. In this case, the adaptive estimation method based on DDPG is no longer applicable to deal with it. Since the target is non-cooperative, the deviation between the process model and the actual model exists, so it is difficult to realize the optimal estimation of measurement noise in a similar way to the adaptive estimation of process noise. To modify the measurement noise adaptively, the recursive estimation of measurement noise is introduced [34].

The estimation of ${\hat{R}}_{k+1}$ can be obtained by maximizing the a posteriori density function, which is given by

(41) $${\hat{R}}_{k+1}=\frac{1}{k+1}\sum_{j=1}^{k+1}\left(z_{j}-h({\hat{x}}_{j})\right){\left(z_{j}-h({\hat{x}}_{j})\right)}^{T}$$

The residual can be represented as

(42) $$\begin{array}{ccc} z_{j}-h({\hat{x}}_{j}) & = & z_{j}-h({\hat{x}}_{j|j-1}+K_{j}\gamma_{j})\\ & = & z_{j}-h({\hat{x}}_{j|j-1})-H_{j}K_{j}\gamma_{j}+ο(K_{j}\gamma_{j})\\ & \approx & \left(I-H_{j}K_{j}\right)\gamma_{j} \end{array}$$

Substitute Equation (42) into Equation (41), yields

(43) $${\hat{R}}_{k+1}=\frac{1}{k+1}\sum_{j=1}^{k+1}\left[\left(I-H_{j}K_{j}\right)\gamma_{j}\gamma_{j}^{T}{\left(I-H_{j}K_{j}\right)}^{T}\right]$$

The expectation of the measurement noise can be expressed as (the detailed derivation can be found in the appendix of [34].)

(44) $$\begin{array}{ccc} E\left[{\hat{R}}_{k+1}\right] & = & E\left\{\frac{1}{k+1}\sum_{j=1}^{k+1}\left(I-H_{j}K_{j}\right)\gamma_{j}\gamma_{j}^{T}{\left(I-H_{j}K_{j}\right)}^{T}\right\}\\ & = & \frac{1}{k+1}\sum_{j=1}^{k+1}\left[R_{j}-\left(I-H_{j}K_{j}\right)H_{j}P_{j|j-1}H_{j}^{T}\right]\\ & = & R-\frac{1}{k+1}\sum_{j=1}^{k+1}\left[\left(I-H_{j}K_{j}\right)H_{j}P_{j|j-1}H_{j}^{T}\right] \end{array}$$

The statistical residual covariance is calculated as the variance of historical residual sequence

(45) $${\bar{R}}_{k+1}=\left(I-H_{k+1}K_{k+1}\right)\left[\gamma_{k+1}\gamma_{k+1}^{T}{\left(I-H_{k+1}K_{k+1}\right)}^{T}+H_{k+1}P_{k+1|k}H_{k+1}^{T}\right]$$

Accordingly, to approximate the real measurement covariance, the modified measurement covariance is updated as the following recursive form

(46) $${\hat{R}}_{k+1}=(1-\rho_{k+1}){\hat{R}}_{k}+\rho_{k+1}{\bar{R}}_{k+1}$$

(47) $$\rho_{k+1}=\frac{1-b}{1-b^{k+1}}$$

where $\rho_{k+1}$ is the weighting factor, and b is a fading factor. The initial value of ${\hat{R}}_{k}$ is the a priori measurement noise covariance matrix.

Therefore, the gain matrix in Equation (6) is replaced as

(48) $$K_{k+1}=P_{k+1|k}H_{k+1}^{T}{\left(H_{k+1}P_{k+1|k}H_{k+1}^{T}+{\hat{R}}_{k+1}\right)}^{-1}$$

By modifying the measurement covariance, the gain matrix is adjusted, then the impact of inaccurate a priori measurement noise is reduced. 

### 4.3. Fusion Algorithm

The local estimations from multiple sensors are fused in this section. Since the cross-covariance matrixes of different sensors are unknown, the fault-tolerant generalized convex combination algorithm (FGCC) [44] based on information theory is introduced in this section.

For two local estimations ${\hat{x}}_{1}$ and ${\hat{x}}_{2}$, the fusion rule of FGCC is

(49) $${\hat{x}}_{f}=P_{f}\left(\upsilon_{1}P_{1}^{-1}{\hat{x}}_{1}+\upsilon_{i}P_{2}^{-1}{\hat{x}}_{2}\right)$$

(50) $$P_{f}={\left(\upsilon_{1}P_{1}^{-1}+\upsilon_{2}P_{2}^{-1}\right)}^{-1}$$

(51) $$\upsilon_{1}+\upsilon_{2}=\delta$$

where $P_{1}$ and $P_{2}$ are corresponding covariance matrices, $\upsilon_{1}$ and $\upsilon_{2}$ are the weighting parameters, and the δ is an adaptive parameter constructed as

(52) $$\delta =\frac{H(P_{1})+H(P_{2})}{H(P_{1})+H(P_{2})+I(P_{1},P_{2})}$$

In the above equation, ℋ is the Shannon entropy, and ℐ is the symmetrized Kullback-Leibler distance between two distributions known as J-divergence.

(53) $$H(P_{i})=\frac{1}{2}\log[{\left(2\pi \right)}^{n}|P_{i}|]+\frac{n}{2}$$

(54) $$I(P_{i},P_{j})=𝒟(P_{i},P_{j})+𝒟(P_{j},P_{i})$$

where n is the number of state vector dimensions, and 𝒟 is the Kullback-Leibler divergence.

(55) $$D\left(P_{i},P_{j}\right)=\frac{1}{2}[\ln\frac{|P_{j}|}{|P_{i}|}+\left|\right|d_{x_{ij}}\left|{|}_{P_{j}^{-1}}+tr\left(P_{i}P_{j}^{-1}\right)-n\right]$$

where $d_{x_{ij}}={\hat{x}}_{i}-{\hat{x}}_{j}$,and $i,j\in \left\{1,2\right\},i\neq j$. 

The weights can be determined by the following equation

(56) $$\upsilon_{i}=\frac{\delta 𝒟(P_{i},P_{j})}{𝒟(P_{i},P_{j})+𝒟(P_{j},P_{i})}$$

Obviously, the above algorithm can only be applied to the fusion of two local estimations. In order to improve the applicability of the fusion algorithm, an extended fault-tolerant generalized convex combination algorithm (EFGCC) is developed, which provides the general fusion rule for N>2.

Suppose there are N local estimations ${\hat{x}}_{i}(i=1,\ldots ,N)$ and corresponding covariance matrices $P_{i}$. The fusion form of EFGCC is given as

(57) $${\hat{x}}_{f}=P_{f}\sum_{i=1}^{N}\upsilon_{i}P_{i}^{-1}{\hat{x}}_{i}$$

(58) $$P_{f}={\left(\sum_{i=1}^{N}\upsilon_{i}P_{i}^{-1}\right)}^{-1}$$

(59) $$\sum_{i=1}^{N}\upsilon_{i}=\delta$$

where the adaptive parameter δ is updated as

(60) $$\delta =\frac{\sum_{i=1}^{N}ℋ\left(P_{i}\right)}{\sum_{i=1}^{N}ℋ\left(P_{i}\right)+\sum_{i=1}^{N}\sum_{j=i+1}^{N}ℐ(P_{i},P_{j})}$$

The definitions of the Shannon entropy and the symmetrized Kullback-Leibler distance are the same as above. The weighting parameter $\upsilon_{i}$ can be determined by the following equation

(61) $$\upsilon_{i}=\frac{\delta f_{i}(𝒟)}{\sum_{j=1}^{N}f_{j}(𝒟)}$$

where

(62) $$\begin{array}{c} f_{i}\left(𝒟\right)=\left\{\begin{array}{cc} 𝒟(P_{i},P_{N})\prod_{j=1}^{i-1}𝒟(P_{N},P_{j})\prod_{j=i+1}^{N-1}𝒟(P_{N},P_{j}) & i<N\\ \prod_{j=1}^{N-1}𝒟(P_{N},P_{j}) & i\geq N \end{array}\right. \end{array}$$

By the fusion rule of Equation (57), the global estimation for maneuvering targets is realized. 

## 5. Simulation

In this section, the effectiveness and superiority of the proposed tracking method are demonstrated through numerical simulations.

### 5.1. Target Model

In the simulation experiments, the Singer model [7] is adopted as the process model in this paper, which describes the target acceleration as the colored noise rather than the white noise. The acceleration is assumed to be a first-order time correlation. The model can be given by

(63) $$x_{k+1}=F_{k+1|k}x_{k}+w_{k}$$

The state vector $x_{k}={\left[x_{k},v_{x,k},a_{x,k},y_{k},v_{y,k},a_{y,k},z_{k},v_{z,k},a_{z,k}\right]}^{T}$, which includes the position, velocity, and acceleration in three directions. 

The state transition matrix is defined as

(64) $$F_{k+1|k}=\mathrm{diag}(F_{x},F_{y},F_{z})$$

where

(65) $$F_{x}=F_{y}=F_{z}=\left[\begin{array}{ccc} 1 & T & (e^{-\alpha T}+\alpha T-1)/\alpha^{2}\\ 0 & 1 & (1-e^{-\alpha T})/\alpha \\ 0 & 0 & e^{-\alpha T} \end{array}\right]$$

α is the target’s maneuvering frequency, and T is the sampling period.

The covariance matrix is calculated as

(66) $$Q_{k}=\mathrm{diag}(Q_{x},Q_{y},Q_{z})$$

where

(67) $$Q_{x}=Q_{y}=Q_{z}=2\alpha \sigma_{a}^{2}{\left[q_{ij}\right]}_{3\times 3}$$

(68) $$\left\{\begin{array}{l} q_{11}=\frac{1}{2\alpha^{5}}\left[1-e^{-2\alpha T}+2\alpha T+2\alpha^{3}T^{3}/3-2\alpha^{2}T^{2}-4\alpha Te^{-\alpha T}\right]\\ q_{12}=q_{21}=\frac{1}{2\alpha^{4}}\left[e^{-2\alpha T}+1-2e^{-\alpha T}+2\alpha Te^{-\alpha T}-2\alpha T+\alpha^{2}T^{2}\right]\\ q_{13}=q_{31}=\frac{1}{2\alpha^{3}}\left[1-e^{-2\alpha T}-2\alpha Te^{-\alpha T}\right]\\ q_{22}=\frac{1}{2\alpha^{3}}\left[4e^{-\alpha T}-3-e^{-2\alpha T}+2\alpha T\right]\\ q_{23}=q_{32}=\frac{1}{2\alpha^{2}}\left[e^{-2\alpha T}+1-2e^{-\alpha T}\right]\\ q_{33}=\frac{1}{2\alpha }\left[1-e^{-2\alpha T}\right] \end{array}\right.$$

The measurement vector is defined as $z_{k}=[r_{k},\beta_{k},\varepsilon_{k}{]}^{T}$, where $r_{k}$ is the range, $\beta_{k}$ is the azimuth angle, and $\varepsilon_{k}$ is the elevation angle. The measurement mapping function is

(69) $$h(x_{k+1})=\left[\begin{array}{l} \sqrt{x_{k+1}{}^{2}+y_{k+1}{}^{2}+z_{k+1}{}^{2}}\\ \arctan(z_{k+1}/x_{k+1})\\ \arctan(y_{k+1}/\sqrt{x_{k+1}{}^{2}+z_{k+1}{}^{2}}) \end{array}\right]$$

The initial covariance matrix of the measurement noise is

(70) $$R=\left[\begin{array}{ccc} \sigma_{r}^{2} & 0 & 0\\ 0 & \sigma_{\beta }^{2} & 0\\ 0 & 0 & \sigma_{\varepsilon }^{2} \end{array}\right]$$

### 5.2. Construction of Neural Networks

**Figure 4:**
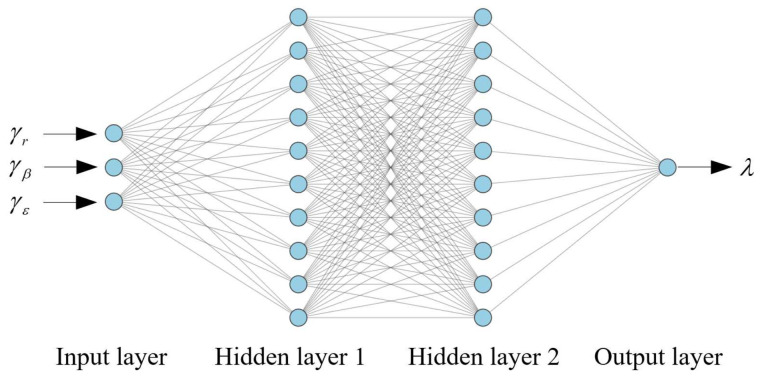
The structure of actor network.

In the proposed process noise estimation based on DDPG, the actor network is used to learn the action policy from the input of the innovation ${\left[\gamma_{k,r},\gamma_{k,\beta },\gamma_{k,\varepsilon }\right]}^{T}$ to the output of the compensation factor $\lambda_{k}$, and the critic network is used to obtain the action-value function based on the innovation and the compensation factor. Inspired by [45,46], four-layer fully connected networks are commonly utilized in deep reinforcement learning applications. Hence the structure of the actor network is designed as shown in Figure 4, and the critic network is represented by the same network structure. Too few neurons will lead to underfitting. Conversely, too many neurons can lead to overfitting and increased training time. By referring to [42,45,46] and simulation experiments, the size of neural networks are set as in Table 1. 

The activation function is tanh function, which is given by

(71) $$\tan h(x)=\frac{e^{x}-e^{-x}}{e^{x}+e^{-x}}$$

Simulation parameters are summarized in Table 2.

The simulation environment is defined as 6000 km×100 km×6000 km, in which the target tracking task is carried out. The training task includes 500 random episodes, and each episode runs 1000 timesteps. The maximum acceleration of the target is 30 m/s^2^, and the minimum acceleration is −30 m/s^2^. For each episode, the target randomly maneuvers with values uniformly distributed between the minimum and the maximum accelerations. According to the definition of the training environment and the maneuver space, training scenarios are constructed.

### 5.3. Simulation Results

To demonstrate the superiority of the proposed filter, IMM [9], STF [22], RNSTF [33], and AKF [34] are simulated for comparison. Two simulation scenarios are conducted in this section to illustrate the feasibility of the proposed noise-adaption EKF. In the first scenario, the target makes continuous time-varying maneuvers. In the second scenario, the target is set to make abrupt maneuvers to verify the performance against strong maneuvers. 

In the simulation, the fading factor in the recursive measurement noise estimation and AKF b=0.9, and the maneuvering frequency in the Singer model α=1/20. The forgetting factor in STF and RNSTF is 0.95. CA model, Singer model, and CS model are adopted in IMM, and the maneuvering frequency in the CS model α=1/20. The initial probability of each sub model is $\left[\begin{array}{ccc} 0.4 & 0.4 & 0.2 \end{array}\right]$, and the Markov transition probability matrix is set as

(72) $$P=\left[\begin{array}{ccc} 0.95 & 0.025 & 0.025\\ 0.025 & 0.95 & 0.025\\ 0.025 & 0.025 & 0.95 \end{array}\right]$$

The root mean square error (RMSE) is generally used to estimate the tracking accuracy, given as

(73) $$\mathrm{RMSE}=\sqrt{\sum_{j=1}^{N_{MC}}{\left({\hat{x}}_{k}^{j}-x_{k}^{j}\right)}^{2}/N_{MC}}$$

where ${\hat{x}}_{k}$ is the estimation value, and $x_{k}$ is the real value. $N_{MC}$ is the number of Monte Carlo experiments, which is 100 in the following simulations.

#### 5.3.1. Continuous Time Varying Maneuver

In this scenario, the target’s acceleration is continuous time-varying in sinusoidal form, which can be described as

(74) $$a=A\sin(\omega t+\varphi_{0})$$

where A is the amplitude, ω is the frequency, and $\varphi_{0}$ is the initial phase. The entire flight process lasts 1000 s. The initial position is [800 km, 80 km, 800 km], and the initial velocity is [−300 m/s, 10 m/s, −300 m/s]. The sinusoidal parameters are set as shown in Table 3.

Assuming that there are three sensors at the origin to track the maneuvering target with a sampling period of 1 s, and a priori measurement errors are set as shown in Table 4. Furthermore, to verify the performance of the proposed noise-adaption EKF under the condition of inaccurate measurement noise, the magnitude of the range measurement noise of $S_{1}$ enlarged five times during 500–600 s, which is unknown to the filter.

**Figure 5:**
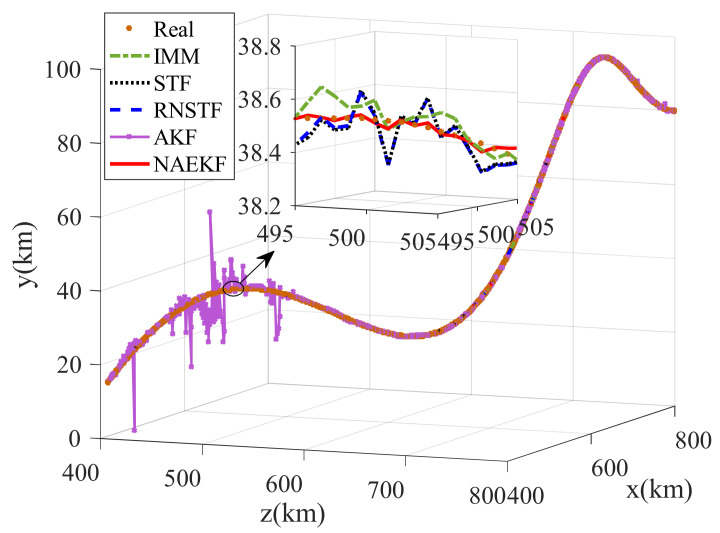
Estimated trajectories.

The algorithms mentioned above are utilized for tracking solution, and the same fusion method, i.e., EFGCC, is adopted to obtain fusion trajectories. For convenience, the proposed noise-adaption EKF is denoted as NAEKF in the following simulation results. The estimated trajectories are shown in Figure 5.

**Figure 6:**
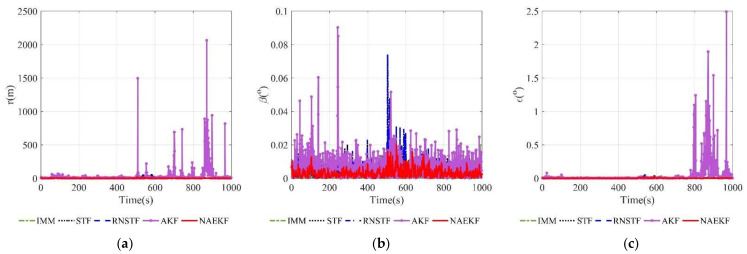
Position estimations errors. (**a**) Estimation errors of the range; (**b**) Estimation errors of the azimuth angle; (**c**) Estimation errors of THE elevation angle.

**Figure 7:**
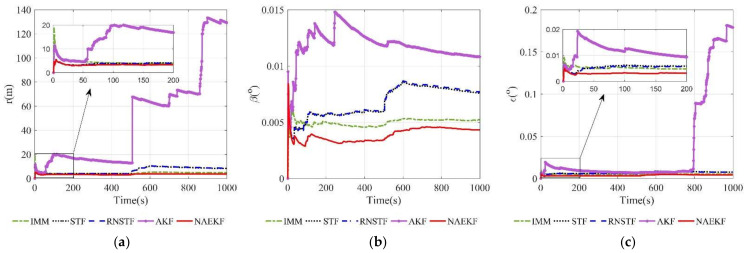
σ-Boundary of position estimation errors. (**a**) σ -Boundary of the range; (**b**) σ -Boundary of the azimuth angle; (**c**) σ -Boundary of the elevation angle.

The position estimation errors are presented in Figure 6. In order to correspond to the measurements, the position estimation errors are given in terms of range, azimuth angle and elevation angle. The position errors of NAEKF are smaller than that of other filters, especially in the stage of inaccurate measurement noise. Correspondingly, Figure 7 shows the σ-boundaries of position estimation errors. At the beginning of the simulation, position errors are generally large due to initialization errors, but the estimation accuracy gradually improves over time. After 100 s, the range error of the proposed filter converges to 3.27 m. The azimuth angle error reaches 0.0033°, and the elevation angle error is stable around 0.0029°. Due to the effect of inaccurate measurement noise between 500–600 s, the stable value of range errors reaches 3.54 m. Similarly, the azimuth angle error increases slightly and finally stabilizes at 0.0043°, and the elevation angle error stabilizes at 0.0043°. To compare local estimation accuracy of different algorithms, the detailed estimation errors are shown in Table 5, Table 6 and Table 7. Taking the range estimation of $S_{1}$ as an example, the estimation accuracy of NAEKF is 8.95%, 21.28% and 20.90% higher than that of IMM, STF and RNSTF, respectively, and the estimation error of AKF is almost seven times that of NAEKF. Obviously, the proposed filter has better tracking accuracy than other filters.

**Figure 8:**
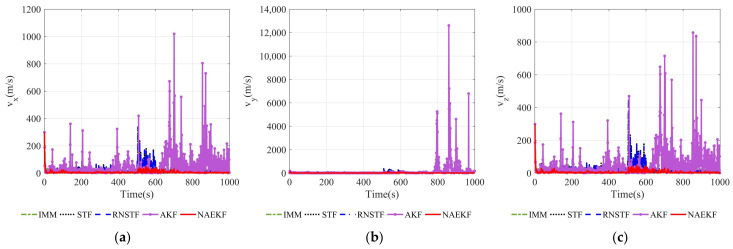
Velocity estimation errors. (**a**) Estimation errors of $v_{x}$; (**b**) Estimation errors of $v_{y}$; (**c**) Estimation errors of $v_{z}$.

**Figure 9:**
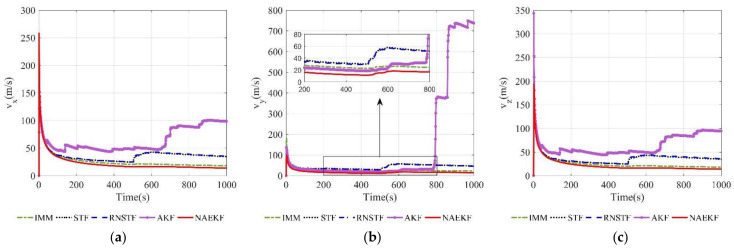
σ-Boundary of velocity estimation errors. (**a**) σ -Boundary of $v_{x}$; (**b**) σ -Boundary of $v_{y}$; (**c**) σ -Boundary of $v_{z}$.

Figure 8 and Figure 9 show the velocity estimation errors and the corresponding σ-Boundary in three directions, respectively. As shown in simulation results, the velocity estimation accuracy of IMM, STF, RNSTF and AKF are worse than that of NAEKF. It can be seen from Figure 9 that the velocity estimation errors of STF and RNSTF increase significantly when inaccurate measurement noise occurs, while the result of IMM and NAEKF do not. Taking the velocity estimation in x direction as an example, the estimation error of STF increases from 24.85 m/s to 35.20 m/s, and that of RNSTF increases from 24.87 m/s to 35.27 m/s. The estimation error of IMM is stable around 18.20 m/s, while the estimation error of AKF reaches 98.65 m/s. In contrast, the velocity estimation error in x direction of NAEKF reaches 13.83 m/s. The tracking results in y and z directions are similar as that in x direction, which will not be repeated here. In the end, the velocity estimation accuracy of NAEKF reaches 14.21 m/s, which is the average of three directions.

**Figure 10:**
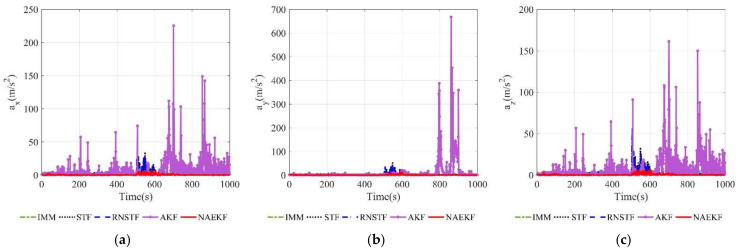
Acceleration estimation errors. (**a**) Estimation errors of $a_{x}$; (**b**) Estimation errors of $a_{y}$; (**c**) Estimation errors of $a_{z}$.

**Figure 11:**
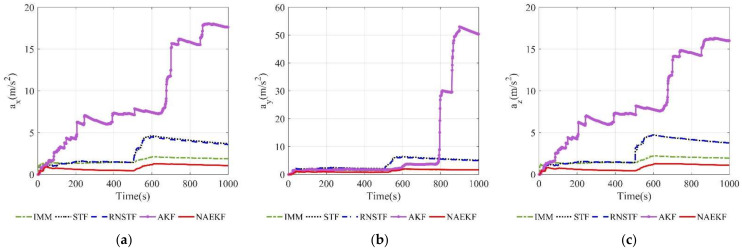
σ-Boundary of acceleration estimation errors. (**a**) σ -Boundary of $a_{x}$; (**b**) σ -Boundary of $a_{y}$; (**c**) σ -Boundary of $a_{z}$.

The acceleration estimation errors and the corresponding σ-boundaries of acceleration estimation errors are presented in Figure 10 and Figure 11, separately. The average acceleration estimation accuracy of NAEKF is 1.27 $m/s^{2}$, whereas that of IMM, STF, RNSTF and AKF is around 1.86 $m/s^{2}$, 4.20 $m/s^{2}$, 4.11 $m/s^{2}$ and 28.01 $m/s^{2}$, respectively. It can be confirmed that the proposed NAEKF can effectively deal with unknown sinusoidal maneuvers and achieves better tracking performance than IMM, STF, RNSTF as well as AKF. Table 8 provides computing times of five algorithms. Since NAEKF adds steps such as maneuver detection, the computing time is slightly longer than that of STF and RNSTF. However, NAEKF takes less computing time than IMM and AKF, because the parallel calculation of sub filters in IMM and the simultaneous noise estimation in AKF are time-consuming.

On the basis of the above experiment settings, the magnitude of the angle measurement noises of $S_{1}$ also enlarged five times during 500–600 s. Simulation results show that the matrix singularity problem of AKF occurs in this simulation experiment. As described in the introduction, AKF shows poor stability, because it updates the process noise and measurement noise simultaneously without distinction. Therefore, IMM, STF, RNSTF and NAEKF are presented in this experiment.

**Figure 12:**
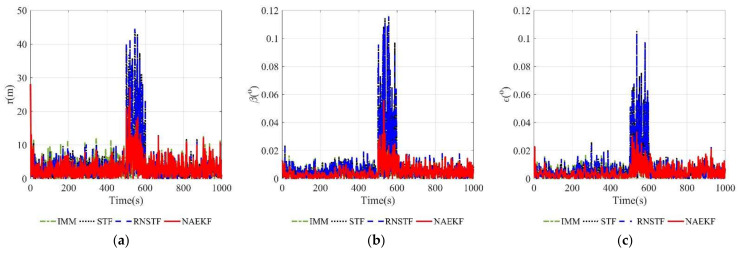
Position estimations errors. (**a**) Estimation errors of the range; (**b**) Estimation errors of the azimuth angle; (**c**) Estimation errors of the elevation angle.

**Figure 13:**
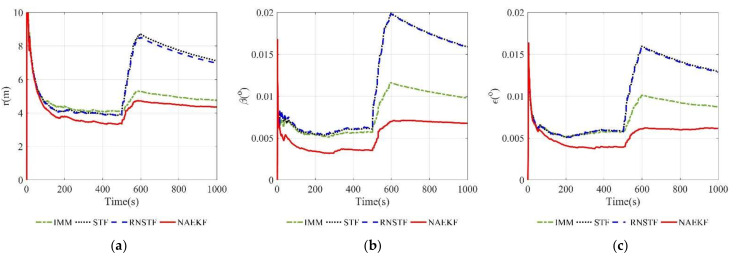
σ-Boundary of position estimation errors. (**a**) σ-Boundary of the range; (**b**) σ-Boundary of the azimuth angle; (**c**) σ-Boundary of the elevation angle.

Figure 12 and Figure 13 show the position estimation errors and the corresponding σ-Boundary. Although the inaccurate measurement noises lead to certain increases in estimation errors, NAEKF shows better estimation accuracy and robustness compared to other three algorithms. Taking the azimuth angle as an example, the estimation error of NAEKF is 0.0068°, while that of IMM reaches 0.0098°. Additionally, the estimation error of STF and RNSTF is 0.0159° and 0.0158°, respectively, which is more than twice the estimation error of NAEKF.

**Figure 14:**
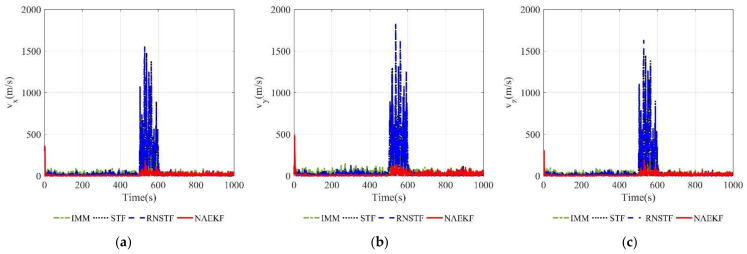
Velocity estimation errors. (**a**) Estimation errors of $v_{x}$; (**b**) Estimation errors of $v_{y}$; (**c**) Estimation errors of $v_{z}$.

**Figure 15:**
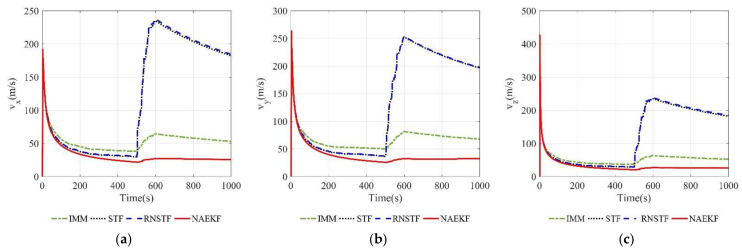
σ-Boundary of velocity estimation errors. (**a**) σ-Boundary of $v_{x}$; (**b**) σ-Boundary of $v_{y}$; (**c**) σ-Boundary of $v_{z}$.

**Figure 16:**
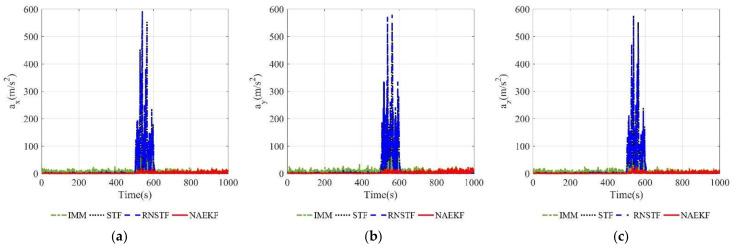
Acceleration estimation errors. (**a**) Estimation errors of $a_{x}$; (**b**) Estimation errors of $a_{y}$; (**c**) Estimation errors of $a_{z}$.

**Figure 17:**
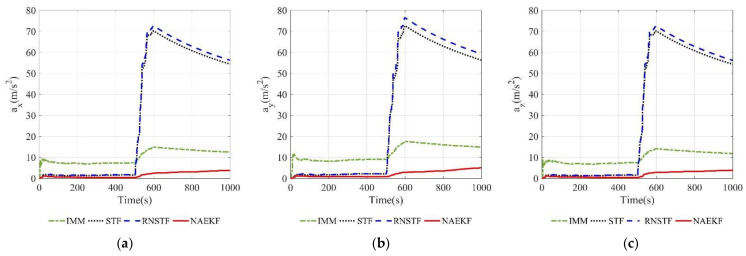
σ-Boundary of acceleration estimation errors. (**a**) σ-Boundary of $a_{x}$; (**b**) σ-Boundary of $a_{y}$; (**c**) σ-Boundary of $a_{z}$.

The estimation results of the velocity and acceleration are shown in Figure 14, Figure 15, Figure 16 and Figure 17. It is obvious that the proposed NAEKF can effectively deal with inaccurate measurement noises in this experiment and achieves the best estimation accuracy compared to other algorithms. The velocity estimation errors of IMM, STF and RNSTF are several times that of NAEKF, and the same conclusion can be drawn from the acceleration estimation results.

#### 5.3.2. Abrupt Maneuver

In this scenario, the target’s acceleration is set to be abrupt, and the maneuver parameters are shown in Table 9. The initial position is [6000 km, 15 km, 6000 km], and the initial velocity is [−1000 m/s, 0 m/s, −1000 m/s].

**Figure 18:**
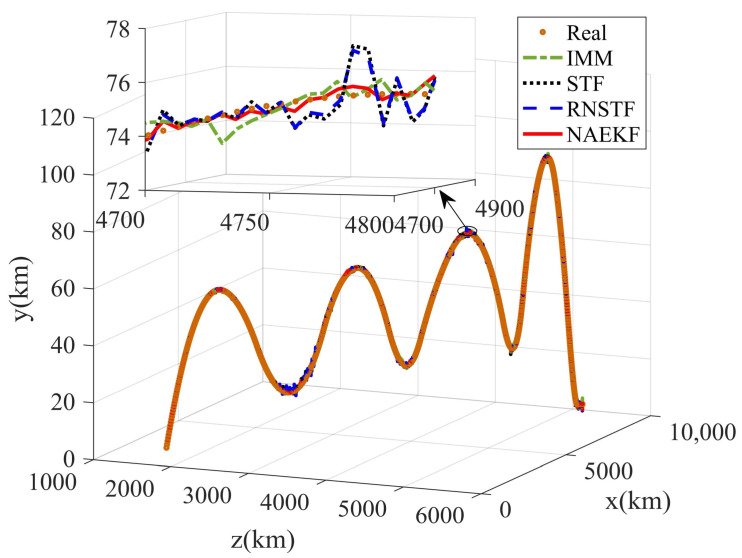
Estimated trajectories.

The measurement errors are the same as in Table 4, and the magnitude of the range measurement noise of $S_{1}$ enlarged five times during 700–800 s. It should be noted that the matrix singularity problem of AKF occurs in this simulation scenario. Therefore, IMM, STF, RNSTF and NAEKF are presented in this section. The tracking results are shown in Figure 18.

**Figure 19:**
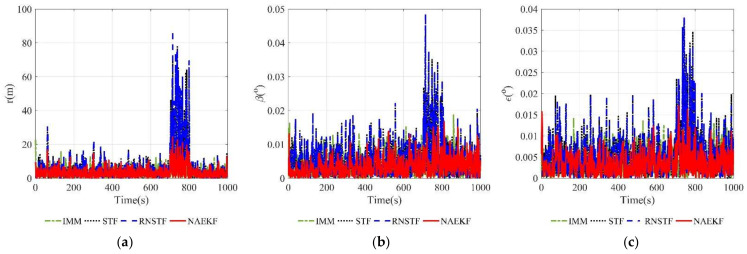
Position estimation errors. (**a**) Estimation errors of the range; (**b**) Estimation errors of the azimuth angle; (**c**) Estimation errors of the elevation angle.

**Figure 20:**
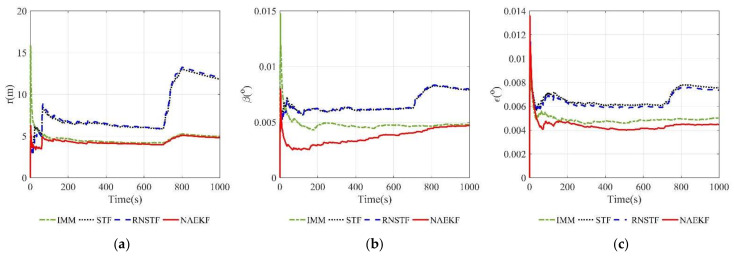
σ-Boundary of position estimation errors. (**a**) σ-Boundary of the range; (**b**) σ-Boundary of the azimuth angle; (**c**) σ-Boundary of the elevation angle.

The position estimation errors and the corresponding σ-boundaries are shown in Figure 19 and Figure 20, respectively. Simulation results show that the proposed filter can track the abrupt maneuver target effectively. Owing to the D-S maneuver detection, the proposed noise-adaption EKF can detect unknown maneuvers and inaccurate measurement noise simultaneously. As a result, the proposed filter can utilize the process noise adaption based on DDPG to adaptively cope with the unknown maneuver. It can be seen from Figure 19a that abrupt maneuvers cause several error increases in the tracking process, but they are quickly eliminated. When inaccurate measurement noise is detected, the proposed filter utilizes the recursive measurement noise adaption to weaken its influence. Certain increases in estimation errors are caused by inaccurate measurement noise during 700–800 s. The estimation errors of STF and RNSTF increase remarkably, especially when the inaccurate measurement noise appears. The estimation errors of different algorithms are shown in Table 10, Table 11 and Table 12. It is evident that the proposed filter achieves higher estimation accuracy than IMM, STF and RNSTF in both local estimation and fusion estimation.

**Figure 21:**
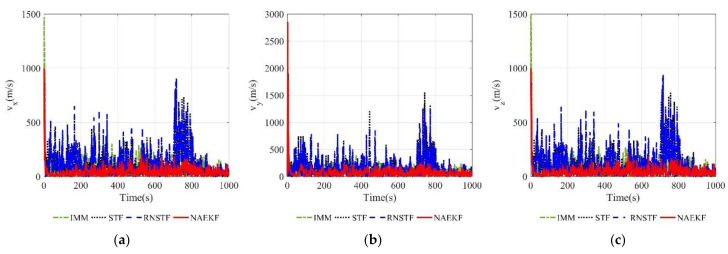
Velocity estimation errors. (**a**) Estimation errors of $v_{x}$; (**b**) Estimation errors of $v_{y}$; (**c**) Estimation errors of $v_{z}$.

**Figure 22:**
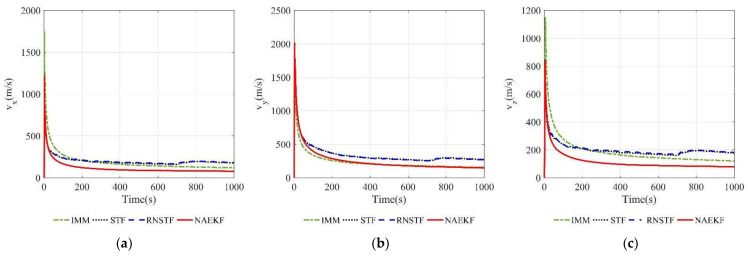
σ-Boundary of velocity estimation errors. (**a**) σ-Boundary of $v_{x}$; (**b**) σ-Boundary of $v_{y}$; (**c**) σ-Boundary of $v_{z}$.

Figure 21 and Figure 22 present velocity estimation errors and corresponding σ-boundaries. Velocity estimation errors in *x* and *z* directions of NAEKF are 77.32 m/s and 77.86 m/s, respectively. However, the estimation error in y direction reaches 146.70 m/s, because the maneuver in y direction is much bigger than other directions. Estimation errors of STF and RNSTF are significantly bigger than that of NAEKF. In contrast, IMM achieves better estimation accuracy than STF and RNSTF, but still worse than NAEKF.

**Figure 23:**
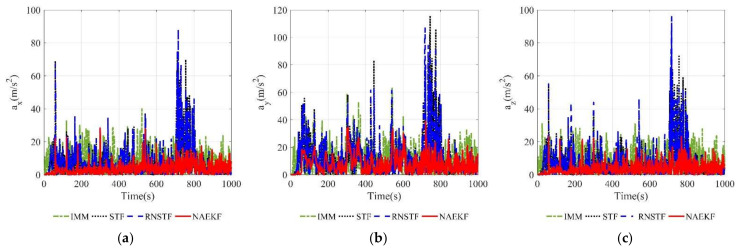
Acceleration estimation errors. (**a**) Estimation errors of $a_{x}$; (**b**) Estimation errors of $a_{y}$; (**c**) Estimation errors of $a_{z}$.

**Figure 24:**
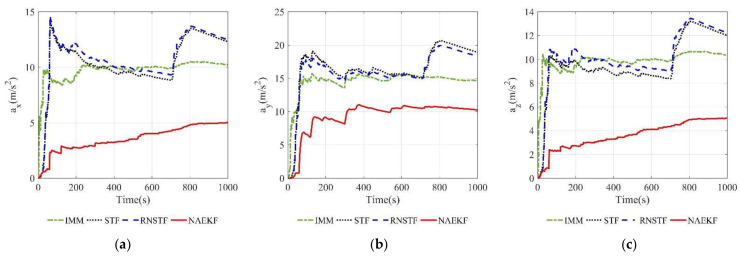
σ-Boundary of acceleration estimation errors. (**a**) σ-Boundary of $a_{x}$; (**b**) σ-Boundary of $a_{y}$; (**c**) σ-Boundary of $a_{z}$.

According to acceleration estimation results in Figure 23, there are several peaks of acceleration tracking errors during abrupt maneuvers, but they decrease rapidly. The σ-boundaries of acceleration estimation errors are shown in Figure 24. The acceleration estimation accuracy in *x* direction of NAEKF is 4.97 $m/s^{2}$, whereas that of IMM, STF and RNSTF is finally stabilized at 10.23 $m/s^{2}$, 12.31 $m/s^{2}$ and 12.49 $m/s^{2}$, respectively. Similarly, the acceleration estimation accuracy in z direction of NAEKF is 5.06 $m/s^{2}$, and the estimation accuracy of IMM, STF and RNSTF is around 10.38 $m/s^{2}$, 12.02 $m/s^{2}$ and 12.24 $m/s^{2}$, respectively. Due to the greater maneuver, the acceleration estimation accuracy in y direction is worse than that in other directions. Specifically, the acceleration estimation error of NAEKF reaches 10.27 $m/s^{2}$. In contrast, the acceleration estimation error of IMM, STF and RNSTF is around 14.66 $m/s^{2}$, 18.93 $m/s^{2}$, and 18.32 $m/s^{2}$, respectively. Table 13 provides computing times of four algorithms. Similarly to the first scenario, the computing time of NAEKF is shorter than that of IMM, but slightly longer than that of STF and RNSTF.

## 6. Conclusions

To address the problem of maneuvering target tracking with inaccurate measurements, the noise-adaption EKF based on DDPG is proposed in this paper. The proposed filter avoids the simultaneous adjustment of the process model and the measurement model without distinction, which effectively improves the robustness of the filter. Within the framework of the noise-adaption EKF, the maneuver detection based on D-S evidence theory is constructed to distinguish between the unknown maneuver and inaccurate measurement noise simultaneously by fusing multi-sensor information. Moreover, a Markovian decision process of maneuver tracking is established to cope with unknown maneuvers. DDPG is developed to learn the adaptive estimation of the compensation factor and feed it to EKF. In addition, the recursive measurement noise estimation is applied to estimate a priori measurement noise covariance online. The local estimations are fused at last, achieving the global estimation of multiple sensors. The simulation results indicate that the proposed noise-adaption EKF is effective in both scenarios of continuous time-varying maneuver and the abrupt maneuver. As shown in simulation results, the proposed tracking method has a better tracking performance compared to IMM, STF, RNSTF and AKF.

## Figures and Tables

**Table 1:** Neural network settings.

Layers	Actor Network	Critic Network
Input layer	3	4
Hidden layer 1	128	128
Hidden layer 2	128	128
Output layer	1	1

**Table 2:** Simulation parameters of DDPG.

Parameters	Value
The discounting factor ξ	0.99
The updating rate τ	0.001
The number of samples $N_{sample}$	64
The number of episodes	500
The number of timesteps	1000

**Table 3:** Sinusoidal parameters.

Directions	x	y	z
A	1	2	1
ω	0.005	0.01	0.005
$\varphi_{0}$	30	30	30

**Table 4:** Measurement accuracy of multiple sensors.

Sensors	$\sigma_{r}\left(m\right)$	$\sigma_{\beta }{(}^{o})$	$\sigma_{\varepsilon }{(}^{o})$
$S_{1}$	10	0.01	0.01
$S_{2}$	5	0.02	0.02
$S_{3}$	10	0.02	0.02

**Table 5:** Estimation errors of the range (m).

Sensors	IMM	STF	RNSTF	AKF	NAEKF
$S_{1}$	15.7454	17.5285	17.4727	96.1808	14.4525
$S_{2}$	4.8372	3.9745	3.9816	3.8560	3.8291
$S_{3}$	8.9003	8.1024	8.1208	7.604	7.3634
Fusion	4.7536	8.1988	8.4076	129.2355	3.5371

**Table 6:** Estimation errors of the azimuth angle (°).

Sensors	IMM	STF	RNSTF	AKF	NAEKF
$S_{1}$	0.0073	0.0098	0.0098	0.0116	0.0057
$S_{2}$	0.0110	0.0130	0.0133	0.0088	0.0090
$S_{3}$	0.0101	0.0135	0.0139	0.0099	0.0087
Fusion	0.0052	0.0076	0.0077	0.0108	0.0043

**Table 7:** Estimation errors of the elevation angle (°).

Sensors	IMM	STF	RNSTF	AKF	NAEKF
$S_{1}$	0.0076	0.0100	0.0098	0.4293	0.0060
$S_{2}$	0.0108	0.0140	0.0136	0.0097	0.0097
$S_{3}$	0.0102	0.0145	0.0139	0.0328	0.0094
Fusion	0.0051	0.0076	0.0074	0.1790	0.0043

**Table 8:** Computing time (s).

IMM	STF	RNSTF	AKF	NAEKF
1.9645	1.7343	1.8173	3.3055	1.8572

**Table 9:** Maneuver parameters of the target.

Time (s)	$\mathrm{Acceleration}\;({m/s}^{2})$			Time (s)	$\mathrm{Acceleration}\;({m/s}^{2})$		
	*x*	*y*	*z*		*x*	*y*	*z*
0–60	0	0	0	480–540	2.5	−5	2.5
60–120	−22.5	18	−22.5	540–600	−12.5	24	−12.5
120–180	2.5	−10	2.5	600–660	2.5	−14	2.5
180–240	−15	−15	−15	660–720	7.5	−10	7.5
240–300	4.5	−10	4.5	720–780	−9	10	−9
300–360	−20	29	−20	780–840	−5.5	11	−5.5
360–420	−10	−8	−10	840–900	4.5	−9	4.5
420–480	−12.5	−10	−12.5	900–1000	2.5	−12	2.5

**Table 10:** Estimation errors of the range (m).

Sensors	IMM	STF	RNSTF	NAEKF
$S_{1}$	18.2491	18.0910	18.2003	17.4784
$S_{2}$	5.0014	5.2004	5.2474	4.6219
$S_{3}$	9.4525	9.2029	9.3168	8.6291
Fusion	4.9532	11.8334	12.0310	4.8331

**Table 11:** Estimation errors of the azimuth angle (°).

Sensors	IMM	STF	RNSTF	NAEKF
$S_{1}$	0.0062	0.0098	0.0099	0.0064
$S_{2}$	0.0110	0.0137	0.0143	0.0104
$S_{3}$	0.0117	0.0133	0.0136	0.0096
Fusion	0.0048	0.0079	0.0080	0.0047

**Table 12:** Estimation errors of the elevation angle (°).

Sensors	IMM	STF	RNSTF	NAEKF
$S_{1}$	0.0062	0.0093	0.0092	0.0057
$S_{2}$	0.0102	0.0141	0.0137	0.0107
$S_{3}$	0.0112	0.0134	0.0131	0.0098
Fusion	0.0050	0.0076	0.0074	0.0045

**Table 13:** Computing time (s).

IMM	STF	RNSTF	NAEKF
1.9920	1.6958	1.8096	1.8723

## Data Availability

Not applicable.
